# Establishment and evaluation of a quadruple quantitative real-time PCR assay for simultaneous detection of human coronavirus subtypes

**DOI:** 10.1186/s12985-022-01793-3

**Published:** 2022-04-11

**Authors:** Mengchuan Zhao, Yi Xu, Dijun Zhang, Guixia Li, Huixia Gao, Xianping Zeng, Yanqing Tie, Yong Wu, Erhei Dai, Zhishan Feng

**Affiliations:** 1 Department of Laboratory Diagnosis, Hebei Medical University, Shijiazhuang, Hebei China; 2 Department of Laboratory Medicine, Children’s Hospital of Hebei Province, Shijiazhuang, Hebei China; 3 Department of Laboratory Medicine, People’s Hospital of Hebei Province, Shijiazhuang, 050031 Hebei China; 4 Department of Laboratory Medicine, The Fifth Hospital of Shijiazhuang, Hebei Medical University, Shijiazhuang, 050031 Hebei China; 5 Ningbo HEALTH Gene Technologies Co., Ltd, Ningbo, Zhejiang China

**Keywords:** Coronavirus, SARS-CoV-2, Subtypes, Quadruple quantitative real-time PCR

## Abstract

**Background:**

The newly discovered severe acute respiratory syndrome coronavirus 2 (SARS-CoV-2) and four seasonal human coronaviruses (HCoVs) (HCoV-229E, HCoV-OC43, HCoV-NL63 and HCoV-HKU1) still circulate worldwide. The early clinical symptoms of SARS-CoV-2 and seasonal HCoV infections are similar, so rapid and accurate identification of the subtypes of HCoVs is crucial for early diagnosis, early treatment, prevention and control of these infections. However, current multiplex molecular diagnostic techniques for HCoV subtypes including SARS-CoV-2 are limited.

**Methods:**

We designed primers and probes specific for the *S* and *N* genes of SARS-CoV-2, the *N* gene of severe acute respiratory syndrome coronavirus (SARS-CoV), and the *ORF1ab* gene of four seasonal HCoVs, as well as the human *B2M* gene product. We developed and optimized a quadruple quantitative real-time PCR assay (qq-PCR) for simultaneous detection of SARS-CoV-2, SARS-CoV and four seasonal HCoVs. This assay was further tested for specificity and sensitivity, and validated using 184 clinical samples.

**Results:**

The limit of detection of the qq-PCR assay was in the range 2.5 × 10^1^ to 6.5 × 10^1^ copies/μL for each gene and no cross-reactivity with other common respiratory viruses was observed. The intra-assay and inter-assay coefficients of variation were 0.5–2%. The qq-PCR assay had a 91.9% sensitivity and 100.0% specificity for SARS-CoV-2 and a 95.7% sensitivity and 100% specificity for seasonal HCoVs, using the approved commercial kits as the reference. Compared to the commercial kits, total detection consistency was 98.4% (181/184) for SARS-CoV-2 and 98.6% (142/144) for seasonal HCoVs.

**Conclusion:**

With the advantages of sensitivity, specificity, rapid detection, cost-effectiveness, and convenience, this qq-PCR assay has potential for clinical use for rapid discrimination between SARS-CoV-2, SARS-CoV and seasonal HCoVs.

**Supplementary Information:**

The online version contains supplementary material available at 10.1186/s12985-022-01793-3.

## Background

There are now seven known human coronaviruses (HCoVs), namely, HCoV-229E, HCoV-OC43, HCoV-NL63, HCoV-HKU1, severe acute respiratory syndrome coronavirus (SARS-CoV), Middle East respiratory syndrome coronavirus (MERS-CoV) and severe acute respiratory syndrome coronavirus 2 (SARS-CoV-2) [1, 2]. Of these, no human infections with the original SARS-CoV have been reported since 2004, and the main risk area for MERS-CoV infections remains the Arabian Peninsula [1]. However, the newly discovered SARS-CoV-2 and four seasonal HCoVs-HCoV-229E, HCoV-OC43, HCoV-NL63 and HCoV-HKU1 still circulate worldwide [2–5]. SARS-CoV-2, first described Wuhan City, China, caused the outbreak of coronavirus disease 2019 (COVID-19) [6–8]. The clinical spectrum of COVID-19 symptoms ranges from asymptomatic or mild flu-like symptoms to fulminant pneumonia with acute respiratory distress syndrome (ARDS) or multiple organ failure resulting in a fatal outcome [9–11]. Thus far, there is no effective treatment for COVID-19. Four seasonal HCoVs cause relatively mild disease accounting for about a third of all “common colds” [3–5, 12], although in infants, the elderly and in immunocompromised patients, they may also be associated with severe lower respiratory tract infections [13, 14]. The early clinical symptoms of SARS-CoV-2 and seasonal HCoV infections are similar, so rapid and accurate identification of HCoV subtypes is crucial to early diagnosis, early treatment, prevention and control of HCoV infections.

At present, laboratory diagnostic techniques for HCoVs include virus culture, immunological assays and molecular testing assays [15]. Virus culture is the “gold standard” of viral diagnosis, but it is not recommended for diagnostic purposes because of time-consuming, complex workflows and strict requirements for the assays. Immunological assays have limitations for use in the early phase of infection because of the time-lag between infection and the development of an adaptive immune response. Molecular testing assays, especially quantitative real-time PCR (qPCR), have been routinely used to detect causative viruses from respiratory secretions, and are currently the gold standard for SARS-CoV-2 detection due to being reliable, fast, high throughput for direct measurement of viral genome constituents rather than secondary biomarkers such as antigens or antibodies [16]. To date, various different molecular diagnostic tests for SARS-CoV-2 have been developed and commercialized, including some kits that can simultaneously detect and distinguish between SARS-CoV-2 and Influenza A, Influenza B, and respiratory syncytial virus [17]. However, few kits or methodologies can simultaneously detect and distinguish between SARS-CoV-2 and other HCoVs.

In the present study, we developed and evaluated a quadruple quantitative real-time PCR assay (qq-PCR) incorporating internal controls in a single closed tube for simultaneous detection of SARS-CoV-2, SARS-CoV and the four seasonal HCoVs HCoV-229E, HCoV-NL63, HCoV-HKU1 and HCoV-OC43. Compared with multiplex PCR based on capillary electrophoresis [18] and sequencing, the most important advantage of our qq-PCR assay is that the amplification and analysis are carried out in a closed system, thus minimizing the possibility of false positive results.

## Methods

### Clinical samples

This study examined a total of 184 clinical samples. Of these, 40 comprised 19 nose swabs, 11 throat swabs and 10 anal swabs from COVID-19 patients and asymptomatic cases at the Fifth Hospital of Shijiazhuang, Hebei Medical University. The remaining 144 were sputum samples collected from inpatients presenting with acute respiratory symptoms at the Children’s Hospital of Hebei Province. The study was conducted with the approval of the Ethics Committee of the Children’s Hospital of Hebei Province and written informed consent was obtained from the patients or in the case of children, from their parents.

### Nucleic acid extraction

Total RNA/DNA was extracted from 200 μL of clinical sample using the TANBead Smart LabAssist-32 extraction system with a TANBead Viral Auto Plate kit (Taiwan Advanced Nanotech Inc., Taoyuan City, Taiwan) according to the manufacturer's instructions. The extracts were eluted in 50 μL of elution buffer and stored at − 20 °C until assayed.

### Primers and probe design

Primers and probes were designed based on the reference sequence (Table 1) of SARS-CoV-2 S and N genes, SARS-CoV N gene, ORF1ab gene of HCoV-229E, HCoV-NL63, HCoV-HKU1 and HCoV-OC43, and human B2M gene using oligo 7.6 with the following parameters: (1) Search Options: TaqMan Probe & PCR pairs; (2) Search Stringency: Very High; (3) Monovalent Ion Concentration: 50.0 mM; (4) Free Mg^2+^ concentration: 3 mM; (5) Primer length: 25 ± 5 bp; (6) Acceptable 3′-Dimer △G: − 2.5 kcal/mol; (7) Primer Tm Range: 60 ± 1 °C; (8) Tm of Probe-Tm of Primer: 5 ± 1 °C; and (9) PCR product length: 80–150 bp. The B2M gene was used as an internal reference for the qq-PCR assay. The sequences and final concentrations of the primers and probes are shown in Table 1. They were synthesized by Sangon Biotech (Shanghai, China).

**Table 1:** Primers and probes of the qq-PCR assay

Targets	Gene	NCBI ID of reference sequence	Genome position	Primer (probe)	Sequence (5′–3′)	Final concentration (μM)
SARS-CoV-2	*S*	NC_045512.2	25,197–25,324	Primer-F1	GGCCATGGTACATTTGGCT	1.2
				Primer-R1	GCAGCAGGATCCACAAGAA	1.2
				Probe 1	FAM-TTGCTGTAGTTGTCTCAAGGGCTG-BHQ-1	0.6
SARS-CoV-2 and SARS	*N*	NC_045512.2	28,677- 28,778	Primer-F2	CTGAGGGAGCCTTGAATACA	1.2
				Primer-R2	TTGGCAATGTTGTTCCTTGA	1.2
				Probe 2	ROX-AACAATGCTGCMAYCGTGCTAC-BHQ-2	0.6
HCoV-229E	*ORF1ab*	NC_002645.1	7464–7568	Primer-F3	ACTTTGTCAGTTCGTATGCTAAAC	1.2
				Primer-R3	TTGTCAGAACATTGGCATTAACA	1.2
				Probe 3	Cy5-ATTATCAGCTTATGACTTGGCGTGT-BHQ-2	0.6
HCoV-NL63	*ORF1ab*	NC_005831.2	23,263–23,376	Primer-F4	GTTCTCTTATAGGTGGCATGGT	1.2
				Primer-R4	GAAGCACATCAGTTTGTAAAGCA	1.2
				Probe 4	Cy5-CCTTTTTCTTTGGCACTGCAAGCAC-BHQ-2	0.6
*HCoV-HKU1*	*ORF1ab*	NC_006577.2	6671–6762	Primer-F5	AGTTTATCTTTAGTTGATGTTTGGGA	1.2
				Primer-R5	GATTTAACTAGGCGTGACAATTCAT	1.2
				Probe 5	Cy5-TATTTGACAGGTTGTGATTATGTTGTTTGGG-BHQ-2	0.6
HCoV-OC43	*ORF1ab*	NC_006213.1	12,518–12,631	Primer-F6	GTGGCCACTAGTTATTATTGCAAA	1.2
				Primer-R6	TCTGGACCACTATTAACAACCTG	1.2
				Probe 6	Cy5-TTGCAAAATAATGAATTAATGCCTGCTAAGTTGA-BHQ-2	0.6
human RNA	B2M	NM_004048.4	92–238	Primer-F7	TCCAGCGTACTCCAAAGATT	1.2
				Primer-R7	CCACTTTTTCAATTCTCTCTCCATT	1.2
				Probe 7	VIC-TCTGCTGGATGACGTGAGTAAACCTG-BHQ-2	0.6

SARS-CoV-2, severe acute respiratory syndrome coronavirus 2; SARS-CoV, severe acute respiratory syndrome coronavirus; HCoV, human coronavirus

### Protocol of the qq-PCR assay

The qq-PCR assay was performed in a final volume of 20 μL containing 5 μL total nucleic acid extracts, 14 μL of reaction mixture and 1 μL of enzyme mixture using a one-step multiplex qRT-PCR on an ABI 7500 (Applied Biosystems, Foster City, USA). The reaction mixture for 50 reactions contained 549 μL of 2 × ZipScript Reaction Buffer I (dNTPs, Mg^2+^ and Buffer) (Qiagen, Hilden, Germany), 12 μL of each of 100 μM forward primer and reverse primer, 6 μL of each of 100 μM probes, 1 μL of 100 μM dUTP Solution (ThermoFisher, Waltham, USA) and 10 μL of RNase-free water. The composition of enzyme mixtures for 50 reactions was 54 μL of 25 × ZipScript Enzyme Mix (Reverse transcriptase and Hot Start DNA Polymerase) (Qiagen, Hilden, Germany) and 6 μL UNG enzyme (ThermoFisher, Waltham, USA). The thermal cycling conditions were as follows: one cycle of 2 min at 25 °C, 30 min at 50 °C, 2 min at 95 °C; 45 cycles of 15 s at 95 °C, 1 min at 60 °C; fluorescent signals were detected at the end of each cycle. Interpretation criteria for the results of the qq-PCR assay are shown in Table 2.

**Table 2:** Interpretation criteria for the results of the qq-PCR assay

Ct value				Result
FAM channel	ROX channel	Cy5 channel	VIC channel	
≥ 38.5/undetermined/no Ct	≥ 38.5/undetermined/no Ct	≥ 38.5/undetermined/no Ct	≤ 38.5	Negative
≤ 38.5	≤ 38.5	≥ 38.5/undetermined/no Ct	≤ 38.5	SARS-CoV-2
≥ 38.5/undetermined/no Ct	≤ 38.5	≥ 38.5/undetermined/no Ct	≤ 38.5	Severe HCoVs (SARS-CoV-2 or SARS-CoV)
≥ 38.5/undetermined/no Ct	≥ 38.5/undetermined/no Ct	≤ 38.5	≤ 38.5	Seasonal HCoVs

Ct, cycle threshold; SARS-CoV-2, severe acute respiratory syndrome coronavirus 2; HCoVs, human coronavirus; SARS-CoV, severe acute respiratory syndrome coronavirus

### Preparation of recombinant plasmids and establishment of standard curves

Recombinant plasmids were constructed using the sequences of the SARS-CoV-2 *S* and *N* genes, the SARS-CoV *N* gene, the *ORF1ab* gene of HCoV-229E, HCoV-NL63, HCoV-HKU1, or HCoV-OC43 or the human *B2M* gene. A section of all target genes was separately amplified with the primers shown in Table 1. The insert size of the SARS-CoV-2 *S* gene was 128 base pairs (bp), the SARS-CoV-2 N gene and the SARS-CoV *N* gene was 102 bp, the HCoV-229E *ORF1ab* gene was 105 bp, the HCoV-HKU1 *ORF1ab* gene was 92 bp, the HCoV-NL63 *ORF1ab* gene was 114 bp, the HCoV-OC43 *ORF1ab* gene was 114 bp, and finally, the human *B2M* gene was 147 bp in size. The PCR products were cloned into the pMD18-T vector, and then transformed into *E. coli*. Picked positive colonies were inoculated into liquid medium and cultured overnight. After PCR and sequencing validation using M13F(-47) primer (CGC CAG GGT TTT CCC AGT CAC GAC) and M13R(-48) primer (AGC GGA TAA CAA TTT CAC ACA GGA), plasmids were extracted using E.Z.N.A Plasmid Miniprep Kit (Omega Bio-tek Inc., Georgia, USA) and quantified by NanoPhotometer N60 (Implen GmbH, München, Germany). The copy number (copies/μL) was calculated using the following equation: [C × 10^–9^ × A/L × 660], in which C represents the concentration of plasmid (ng/μL) assessed by the optical density measurement; A is the Avogadro number (6.023 × 10^23^); L is the length of the plasmid (number of nucleotides); and 660 is an approximation of the molecular weight of a nucleotide (g/mol).

The recombinant plasmids were used as standards for the quantitative analysis of the qq-PCR assay. The qq-PCR standard curves were individually generated for each target gene by serial tenfold dilutions of the eight recombinant plasmids with a known copy number from 10^1^ to 10^8^ copies/μL.

### Analytical sensitivity, specificity and reproducibility of qq-PCR assay

Ten-fold dilutions of recombinant plasmid from 10^2^ to 10^6^ copies/μL were used as the standard to quantify the sensitivity of the qq-PCR assay, with 20 replicates at each concentration. Detection rates at different concentrations were summarized for probabilistic analysis, and the minimum recombinant plasmid concentration yielding a detection rate of 95% (at least 19 times in 20 replicates) was taken as the limit of detection (LOD). The specificity of the qq-PCR assay was evaluated using mixed recombinant plasmids containing the sequences of the SARS-CoV-2 S and N genes, the SARS-CoV N gene, the ORF1ab gene of HCoV-229E, HCoV-NL63, HCoV-HKU1 and HCoV-OC43, and the human B2M gene, as well as 11 other common respiratory pathogen-positive nucleic acid samples retrospectively confirmed by a commercial kit (Respiratory Pathogen 13 Detection Kit (Multiplex-PCR) (Health Gene Technologies, Ningbo, China)). Negative control (RNase-free water incorporating internal reference), low concentration plasmid [(2 ~ 3) × LOD] and moderately concentrated plasmid [(5 ~ 7) × LOD] were tested by two different technicians using three batches of reagents for the intra-assay coefficient of variation (CV) of this assay. These simulated specimens were also tested by two technicians on five different days within one week in order to establish inter-assay reproducibility.

### Clinical performance of the qq-PCR assay

The reference kits in this study were the 2019-nCoV Detection Kit (qPCR) (Daan Gene, Guangzhou, China) for SARS-CoV-2 and the Respiratory Pathogen 13 Detection Kit (Multiplex-PCR) (Health Gene Technologies, Ningbo, China) for four seasonal HCoVs. The *ORF1ab* and *N* genes are the targets used for SARS-CoV-2 detection by the 2019-nCoV Detection Kit (qPCR). The Respiratory Pathogen 13 Detection Kit can simultaneously detect Influenza A, Influenza A H1N1 pdm09, Influenza H3N2, Influenza B, human parainfluenza virus, respiratory syncytial virus, human adenovirus, human metapneumovirus, rhinovirus, human bocavirus, seasonal HCoVs, Chlamydia and Mycoplasma pneumoniae. These two test kits are approved by NMPA.

A total of 184 clinical samples was assessed by the qq-PCR assay and the 2019-nCoV Detection Kit (qPCR) according to the protocol of the qq-PCR assay or the manufacturer's instructions in order to evaluate the clinical performance of the qq-PCR assay for SARS-CoV-2. Sputum samples from inpatients presenting with acute respiratory symptoms (n = 144) were also tested using the Respiratory Pathogen 13 Detection Kit to evaluate clinical performance of the qq-PCR assay for seasonal HCoVs.

When results were discordant between the qq-PCR assay and reference kits, both tests were repeated concurrently to evaluate any problems relating to potential hands-on error. If still discordant, that sample was investigated by Sanger sequencing.

### Statistical analysis

Statistical analyses were performed using Statistical Product and Service Solutions (SPSS) 21.0 software (IBM, Armonk, NY, USA). The results from reference kits and the qq-PCR assay were analyzed using kappa and McNemar tests, and the level of statistical significance was set at *P* < 0.05. Kappa stands for the measure of agreement between the two tests; a value of > 0.9 is excellent. Statistical Analyses of the CV value of the qq-PCR assay were performed according to CLSI EP5-A2 guidelines.

## Results

### Sensitivity

**Fig. 1:**
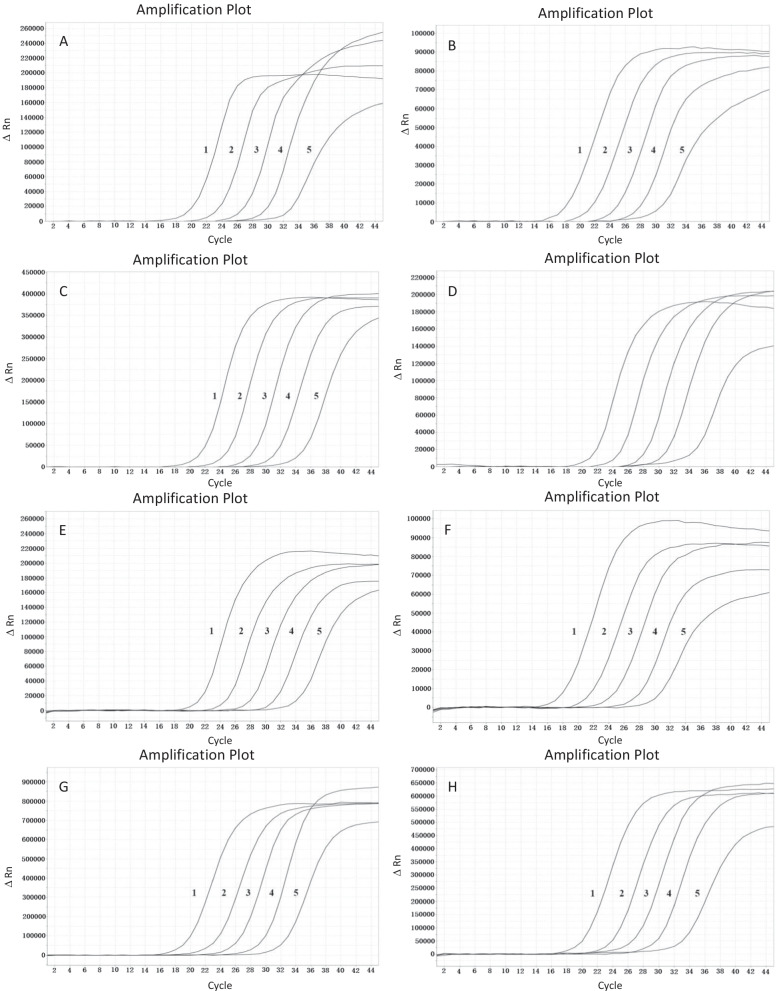
The sensitivity analysis results of the qq-PCR assay. **A**–**H** Amplification curves of the SARS-CoV-2 S gene, SARS-CoV-2N gene, SARS-CoV N gene, HCoV-229E ORF1ab gene, HCoV-NL63 ORF1ab gene, HCoV-HKU1 ORF1ab gene, HCoV-OC43 ORF1ab gene and human RNA B2M gene using tenfold serial dilutions of the recombinant plasmids from 10^6^ to 10^2^ copies/μl

The results of the sensitivity analysis are shown in Fig. 1 and Table 3. The LOD of the qq-PCR assay for each gene ranged from 2.5 × 10^1^ to 6.5 × 10^1^ copies/μL (Table 3). The standard curves of the qq-PCR assay were linear over the range 10^2^–10^6^ copies/μl for each gene and the correlation coefficients (R2) for each were between 0.997 and 0.999 (Table 3), indicating that the assay was accurate, precise and stable over this range.

**Table 3:** The correlation coefficients and limit of detection for each gene

Targets	Gene	Correlation coefficients	Limit of detection (copies/μl)
SARS-CoV-2	*S*	0.998	3.2 × 10^1^
SARS-CoV-2	*N*	0.999	4.8 × 10^1^
SARS-CoV	*N*	0.998	4.8 × 10^1^
HCoV-229E	*ORF1ab*	0.999	3.8 × 10^1^
HCoV-NL63	*ORF1ab*	0.997	6.5 × 10^1^
HCoV-HKU1	*ORF1ab*	0.998	4.2 × 10^1^
HCoV-OC43	*ORF1ab*	0.999	3.7 × 10^1^
human RNA	*B2M*	0.997	2.5 × 10^1^

SARS-CoV-2, severe acute respiratory syndrome coronavirus 2; SARS-CoV, severe acute respiratory syndrome coronavirus; HCoV, human coronavirus

### Specificity

Mixed recombinant plasmids containing the sequences of the SARS-CoV-2 S and N genes, the SARS-CoV N gene, the ORF1ab gene of HCoV-229E, HCoV-NL63, HCoV-HKU1 and HCoV-OC43, and the human B2M gene were tested by the qq-PCR assay. The cycle threshold (Ct) value of the FAM, ROX, CY5 and VIC channels were all ≤ 38.5 (data not shown). A total of 11 nucleic acid samples confirmed positive for Influenza A, Influenza B, human parainfluenza virus, respiratory syncytial virus, human adenovirus, human metapneumovirus, rhinovirus, human bocavirus, Chlamydia pneumoniae, Chlamydia trachomatis or Mycoplasma pneumoniae was also tested by the qq-PCR assay. All samples yielded a negative result, suggesting that the qq-PCR assay did not exhibit any cross-reactivity with any of these pathogens (data not shown).

### Reproducibility

The results of the reproducibility analysis are shown in Additional file 1: Tables S1 and S2. The CV values of the low concentration plasmid in intra-assay and inter-assay variability assessments were < 2%, and those for the moderately concentrated plasmid were < 1.5%. No target genes were detected in the negative control and the internal reference was as expected, both with CV values < 0.5%.

### Clinical performance of the qq-PCR assay

Of the 184 clinical samples, 34 were SARS-CoV-2-positive (Table 4) and 45 were seasonal HCoV-positive by the qq-PCR assay (Table 5), whereas 37 were SARS-CoV-2-positive using the 2019-nCoV Detection Kit (qPCR) (Table 4) and 47 were seasonal HCoV-positive with the Respiratory Pathogen 13 Detection Kit (Multiplex-PCR) (Table 5). Thus, the qq-PCR assay diagnostic sensitivity and specificity of SARS-CoV-2 against the 2019-nCoV Detection Kit (qPCR) as the reference was 91.9% and 100.0%, respectively (Table 4). A sensitivity of 95.7% and a specificity of 100.0% for the qq-PCR assay of seasonal HCoVs against the Respiratory Pathogen 13 Detection Kit (Multiplex-PCR) as the reference was recorded. The consistency for SARS-CoV-2 between the qq-PCR assay and the 2019-nCoV Detection Kit (qPCR) was 98.4%, with a Kappa value of 0.948 (*P* < 0.01) (Table 4); for seasonal HCoVs it was 98.6% between the qq-PCR assay and the Respiratory Pathogen 13 Detection Kit (Multiplex-PCR), with Kappa value of 0.968 (*P* < 0.01) (Table 5).

**Table 4:** The clinical performance of the qq-PCR assay for SARS-CoV-2

	2019-nCoV qPCR		Performance characteristics						
	Positive	Negative	Sensitivity (%)	Specificity (%)	Youden's Index	PPV (%)	NPV (%)	Agreement	Kappa
qq-PCR assay									
Positive	34	0	91.9	100.0	0.919	100.0	98.0	98.4	0.948 (*P* < 0.01)
Negative	3	147							
Total	37	147							

SARS-CoV-2, severe acute respiratory syndrome coronavirus 2; PPV, positive predictive value; NPV, negative predictive value

**Table 5:** The clinical performance of the qq-PCR assay for seasonal HCoVs

	Respiratory pathogen 13 detection Kit		Performance characteristics						
	Positive	Negative	Sensitivity (%)	Specificity (%)	Youden's Index	PPV (%)	NPV (%)	Agreement	Kappa
qq-PCR assay									
Positive	45	0	95.7	100.0	0.957	100.0	98.0	98.6	0.968 (*P* < 0.01)
Negative	2	97							
Total	47	97							

HCoVs, human coronavirus; PPV, positive predictive value; NPV, negative predictive value

Of the 184 samples tested, the qq-PCR assay results of 5 were inconsistent with the reference kit results. Three of the 5 samples had a Ct value > 38.00 by the 2019-nCoV Detection Kit (qPCR) but were missed by the qq-PCR assay. The remaining two were negative by the qq-PCR assay but positive for seasonal HCoVs by the Respiratory Pathogen 13 Detection Kit (Multiplex-PCR). All these positive samples with discordant results were confirmed by sequencing as true positives.

## Discussion

HCoVs are important respiratory pathogens. There are four species of seasonal HCoVs-HCoV-NL63, HCoV-229E, HCoV-OC43 and HCoV-HKU1-that are usually associated with relatively mild respiratory symptoms and ranging in frequency from 0.3 to 4.5% [19–21]. SARS-CoV-2, a novel coronavirus responsible for an ongoing pandemic, has had a significant impact on both public health and the economy worldwide [2]. The manifestations of SARS-CoV-2 infection are highly nonspecific with the most common symptoms including fever, cough and fatigue, similar to the clinical symptoms of seasonal HCoV infections [22, 23]. A large number of commercial kits have been developed for specifically detecting SARS-CoV-2 [17], but methods to simultaneously detect SARS-CoV-2 and other HCoVs only are available via sequencing and a multiplex PCR assay based on capillary electrophoresis [18]. For these approaches, it is necessary to open the reaction tube again after amplification to transfer the amplification products, which increases the risk of contamination. Moreover, both of these methodologies are labor-intensive and time-consuming, indicating a need for a rapid, accurate and high-throughput approach to HCoV subtype testing.

Quantitative real-time PCR (qPCR) with high specificity and sensitivity is sufficiently reliable and is a fast technique. It facilitates the analysis of the results in real time even when the process is still ongoing, and the single-tube design reduces the possibility of cross-contamination. In addition, the development of multiple qPCR improves the detection throughput and offers the possibility to study multiple targets [24, 25]. These advantages enable qPCR, a widely deployed technique in diagnostic virology, to become an increasingly important laboratory method for detecting, tracking, and studying infectious disease pathogens [26–29]. In the present study, for rapid, simple and sensitive differential detection of SARS-CoV-2, SARS-CoV and four seasonal HCoVs, we successfully established an internally controlled qq-PCR assay in a single closed tube based on the qPCR technique.

To this end, we used recombinant plasmids as the standards for quantitative analysis of our qq-PCR assay. Although this type of experiment should ideally have been carried out with in vitro transcription of RNA, recombinant plasmids possess better stability for storage and are more convenient for quantification than cDNA used for the in vitro transcription. Regarding the criteria for interpreting the results of the qq-PCR assay, the cut-off point was first set at Ct value 38 based on our past experience [24], after which it was adjusted to 38.5 by comparing the results from 184 clinical samples between the qq-PCR assay and reference kits.

The qq-PCR assay has a 91.9% sensitivity and 100.0% specificity for SARS-CoV-2 and 95.7% sensitivity and 100% specificity for seasonal HCoVs. Compared to the two commercial kits, the detection consistency was 98.4% with a 0.948 Kappa value (*P* < 0.01) for SARS-CoV-2 and 98.6% with a 0.968 Kappa value (*P* < 0.01) for seasonal HCoVs, indicating that the clinical performance of the qq-PCR assay is similar to the approved commercial kits.

Four samples missed by qq-PCR assay either had high Ct values (positive only by the 2019-nCoV Detection Kit) or a weak fluorescence signal (positive only by the Respiratory Pathogen 13 Detection Kit), we speculated that these samples had a low virus titer that is below the LOD of the qq-PCR. One other inconsistent sample (positive only by the Respiratory Pathogen 13 Detection Kit) with an atypical S-shaped amplification curve contains a mutation in the probe-covered area, which led to the false negative result. Although the qq-PCR assay is slightly less sensitive than the two approved commercial kits, its LODs ranged from 2.5 × 10^1^ to 6.5 × 10^1^ copies/μL for each gene, suggesting that it had sufficient sensitivity and would be adequate for the differential diagnosis of HCoV infections. In addition, the qq-PCR assay exhibited stable reproducibility and no cross-reactivity with other common respiratory pathogens. Moreover, unlike the sequencing and multiplex PCR assay based on capillary electrophoresis [18], the qq-PCR assay incorporates an internal reference gene for quality control of the entire detection process including extraction, reverse transcription and amplification, contributing to the avoidance of false-negative results. Reciprocally, the UNG enzyme and dUTP were used to effectively prevent the contamination of amplification products and avoid false positive results. Thus, the qq-PCR assay developed in this study is highly suitable for HCoV typing to meet the challenge of clinical diagnosis and with advantage of low cost and rapidity.

A limitation of the qq-PCR assay is that although it detects four seasonal HCoVs it cannot distinguish between them. Additionally, the behavior of this assay in the face of a specific target was evaluated only by mixed recombinant plasmids, and further evaluation using mixed clinical samples including both seasonal HCoVs and SARS-CoV-2 is needed for its validation.

In conclusion, we have developed a qq-PCR assay incorporating internal controls in a single closed tube for combined detection of SARS-CoV-2, SARS-CoV and four seasonal HCoVs. The qq-PCR assay offers the advantages of high sensitivity and specificity, rapid detection, cost-effectiveness and convenience, and should be of great interest for routine clinical use.

## Supplementary Information

**Additional file 1:** Inter-assay and Intra-assay of coefficient of variation (CV) of the qq-PCR assay.

## Data Availability

The datasets used and/or analyzed during the current study available from the corresponding author on reasonable request.

## Abbreviations

SARS-CoV-2: Severe acute respiratory syndrome coronavirus 2
HCoVs: Human coronaviruses
SARS-CoV: Severe acute respiratory syndrome coronavirus
qq-PCR: Quadruple quantitative real-time PCR assay
CV: Coefficients of variation
MERS-CoV: Middle East respiratory syndrome coronavirus
COVID-19: Coronavirus disease 2019
ARDS: Acute respiratory distress syndrome
qPCR: Quantitative real-time PCR
Ct: Cycle threshold
bp: Base pairs
LOD: Limit of detection
R2: The correlation coefficients
